# Supplementary material for: Outstanding TC Enhancement in 5d–3d Y2NiIrO6 by Compression
Source: Adv Sci (Weinh). 2026 May 21:e75739. Online ahead of print. doi: 10.1002/advs.75739 (PMC13335984; doi:10.1002/advs.75739)
Supplement: Supplementary file 1 — Supporting File: advs75739‐sup‐0001‐SuppMat.docx. [file ADVS-9999-e75739-s001.docx]

Supplementary Materials for

**Outstanding *T_C_* Enhancement in 5*d*–3*d* Y_2_NiIrO_6_ by Compression**

Zheng Deng^a,^*, Yao Zhang^b^, Sijia Zhang^a^, Jing Song^a^, Wanli He^c^, Yuanzhe Li^c^, Meilin Jin^c^, Xiang Li^c^, Guanghua Liu^d^, Zhen Dong^d^, Jinkai Bi^d^, Wenmin Li^d^, Jianfa Zhao^a^, Jun Zhang^a^, Yi Peng^a^, Luchuan Shi^a^, Junling Meng^b,^*, Xiancheng Wang^a^, Changqing Jin^a,^*

*^a^ Beijing National Laboratory for Condensed Matter Physics, Institute of Physics, Chinese Academy of Sciences and School of Physics, University of Chinese Academy of Sciences, Beijing, 100190, China*

*^b^ School of Chemistry, Jilin Normal University, Changchun, 130024, China*

*^c^ Centre for Quantum Physics, Key Laboratory of Advanced Optoelectronic Quantum Architecture and Measurement (MOE) and School of Physics, Beijing Institute of Technology, Beijing, 100081, China*

*^d^ Institute of Quantum Materials and Physics, Henan Academy of Sciences, Zhengzhou, 450046, China*

Correspondence to: Z. Deng (dengzheng@iphy.ac.cn), J. Meng (mengjunling@jlnu.edu.cn), C. Jin (jin@iphy.ac.cn)

**Figure S1**

**Figure S1. *In-situ* synchrotron XRD from ambient pressure to 35.0 GPa.**

**Figure S2**


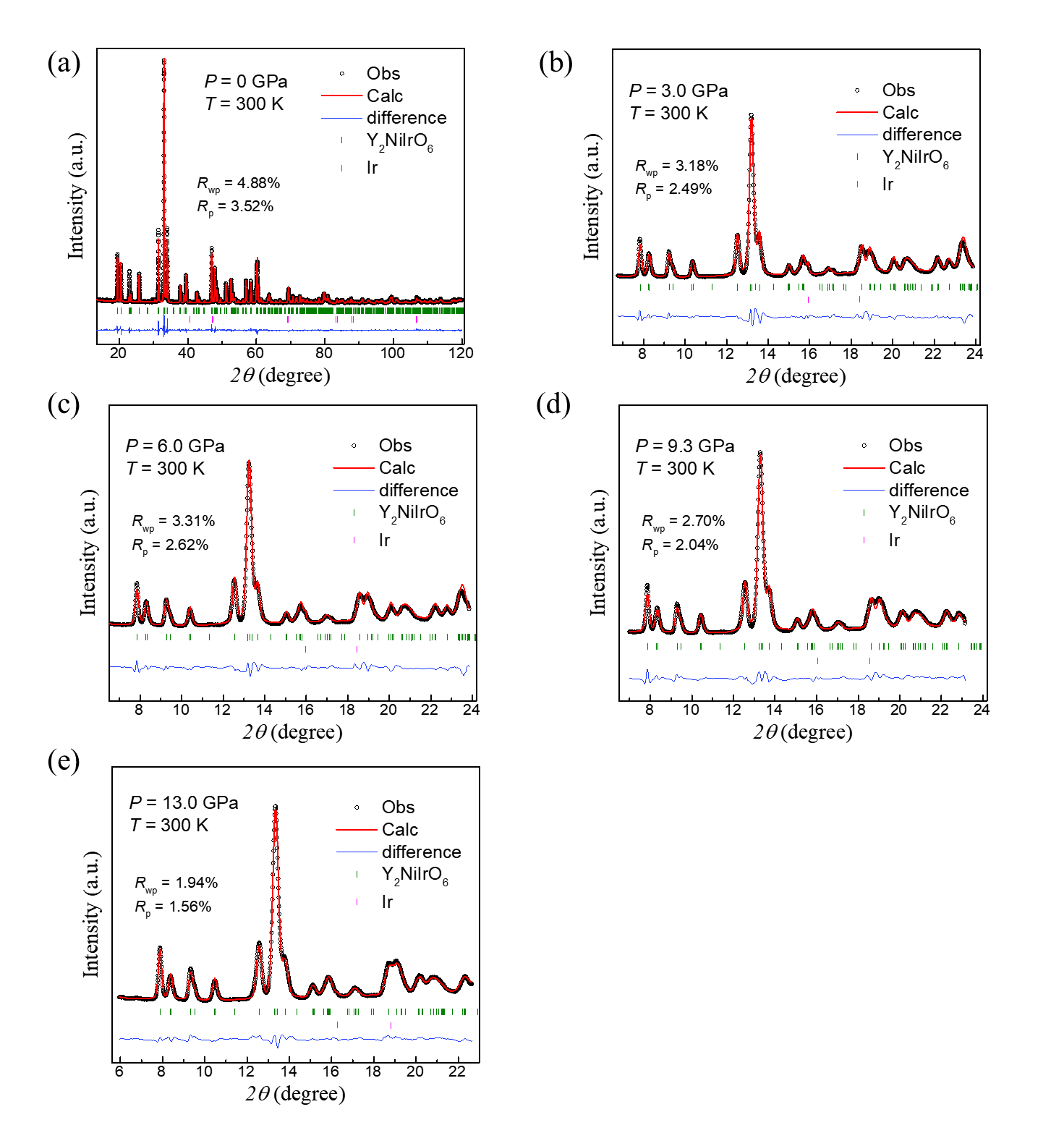


**Figure S2. (a)-(e) Rietveld refinements of XRD in Figure S1 under varying pressures.**

**Figure S3**

Raman spectrum is a good supplementary method to probe crystal structural transitions. A double perovskite material with space group of P2_1_/n has 24 Raman-active modes, but several of them cannot be observed because of the accidental degeneracy. Figure S1 shows the Raman spectra of Y_2_NiIrO_6_ at pressures from 0 to 31.6 GPa. About 10 bands can be observed between 150 and 850 cm^-1^ at ambient pressure. Here, we choose five unambiguous ones as A1, A2, A3, A4, and A5 which are locate at 180, 219, 329, 553, and 667 cm^−1^ respectively. All the peaks gradually move to larger wave number upon compassion as shown in Figure S3. Above 17 GPa, one can find a new band at 598 cm^−1^, which is labeled as modes A6. The presence of the new band indicates the structural phase transition. The transition pressure is consistent with the XRD results. After decompression to ambient pressure, the Raman spectrum of low-pressure phase can be recovered.

**Figure S3. Raman spectra of Y_2_NiIrO_6_ at pressures from 0 to 31.6 GPa.**

**Figure S4**

**
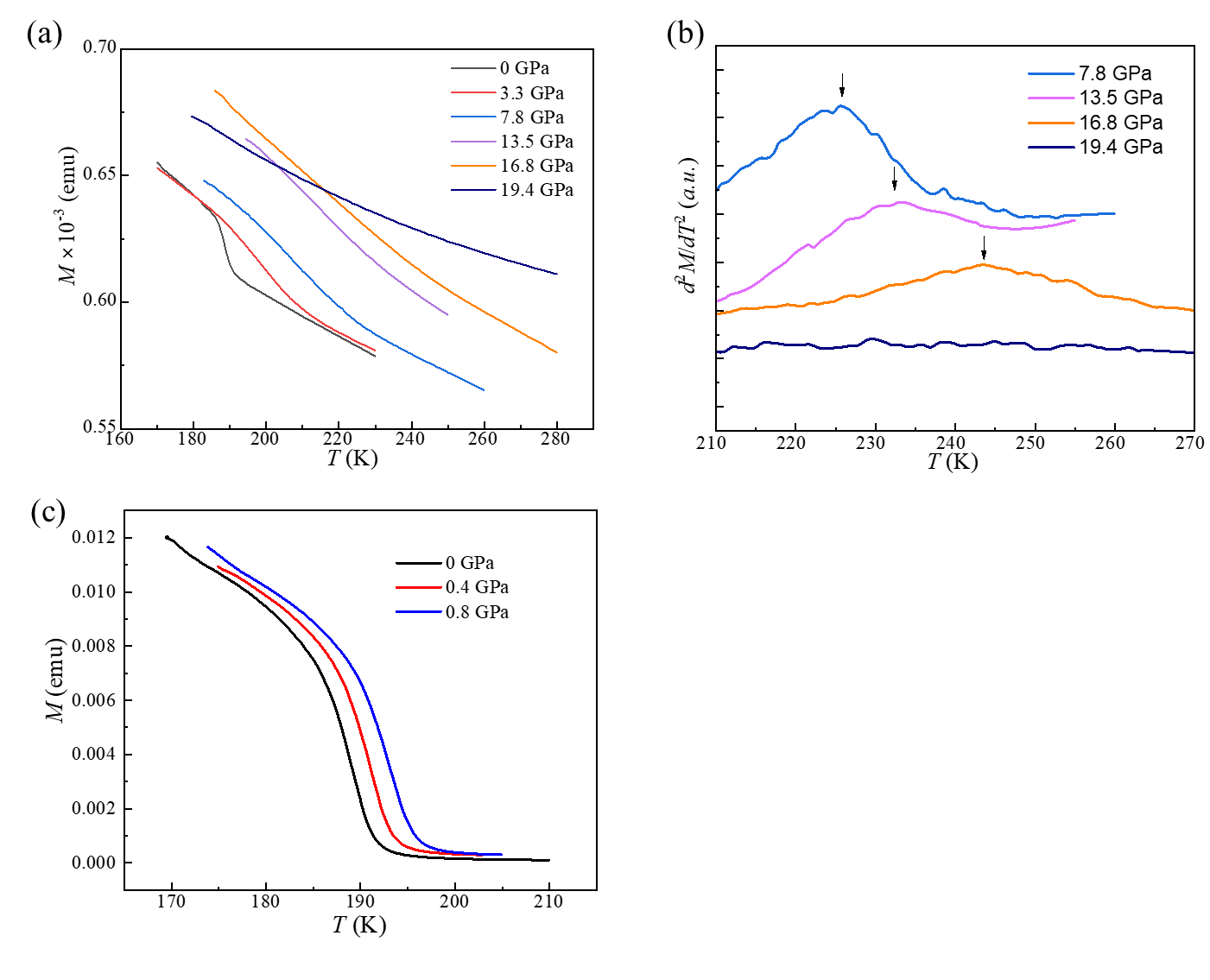
**

**Figure S4.** (a) Raw data of temperature-dependent magnetization (*M*(*T*)) measured with DAC. (b) The second derivative of *M*(*T*) curve. The peaks indicate the change of slope on *M*(*T*) and corresponding to the forming of magnetic order. (c) Raw data of *M*(*T*) measured with the piston-cylinder cell.

**Figure S5**

To clarify the effect of pressure on magnetocrystalline anisotropy (MCA), we measured *M*(*H*) using a piston-cylinder cell and focus on the evolution of *H*_C_, *H*_EB_, and *M*_shift_ with increasing pressure. As established in Ref. 19 (main text), *H*_EB_, and *M*_shift_ originate from the large MCA in YNIO, while *H*_C_ itself reflects the strength of MCA. All three parameters increase under compression across different cooling fields (Figure S5, Table S2), indicating that MCA is enhanced by pressure.

**
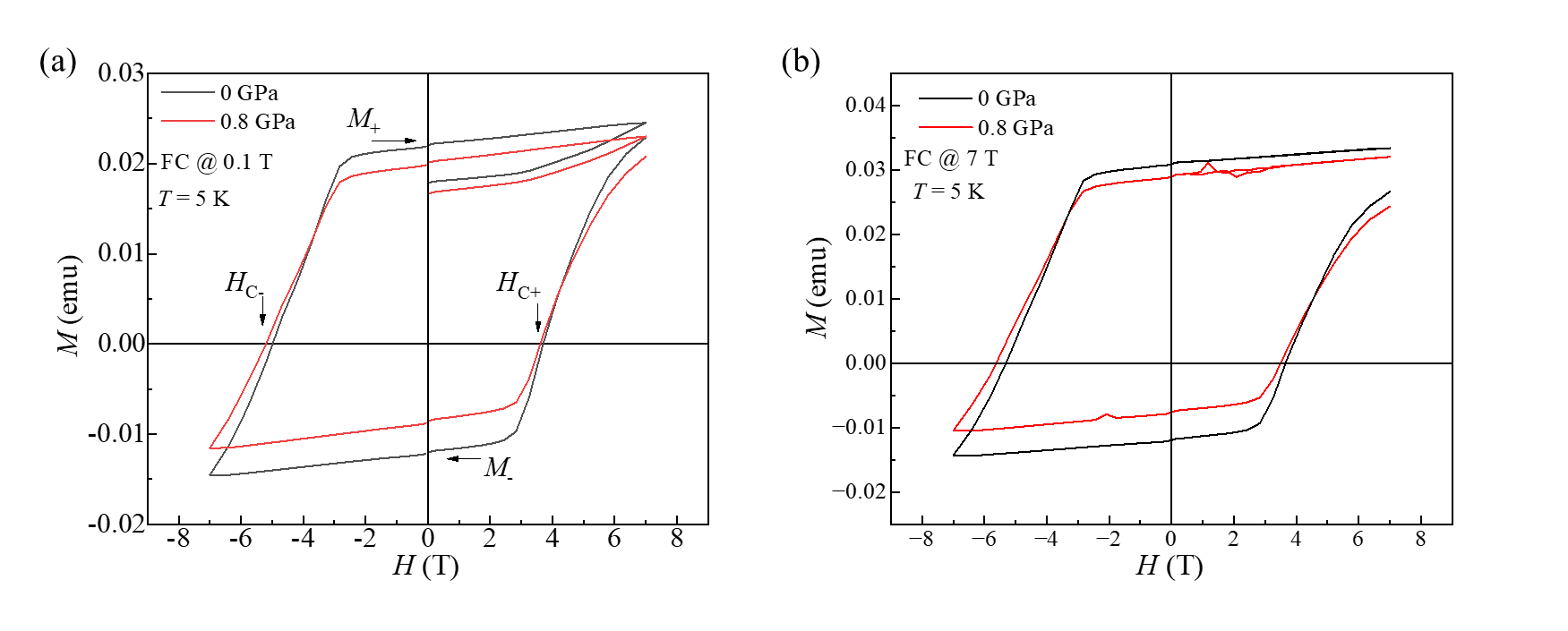

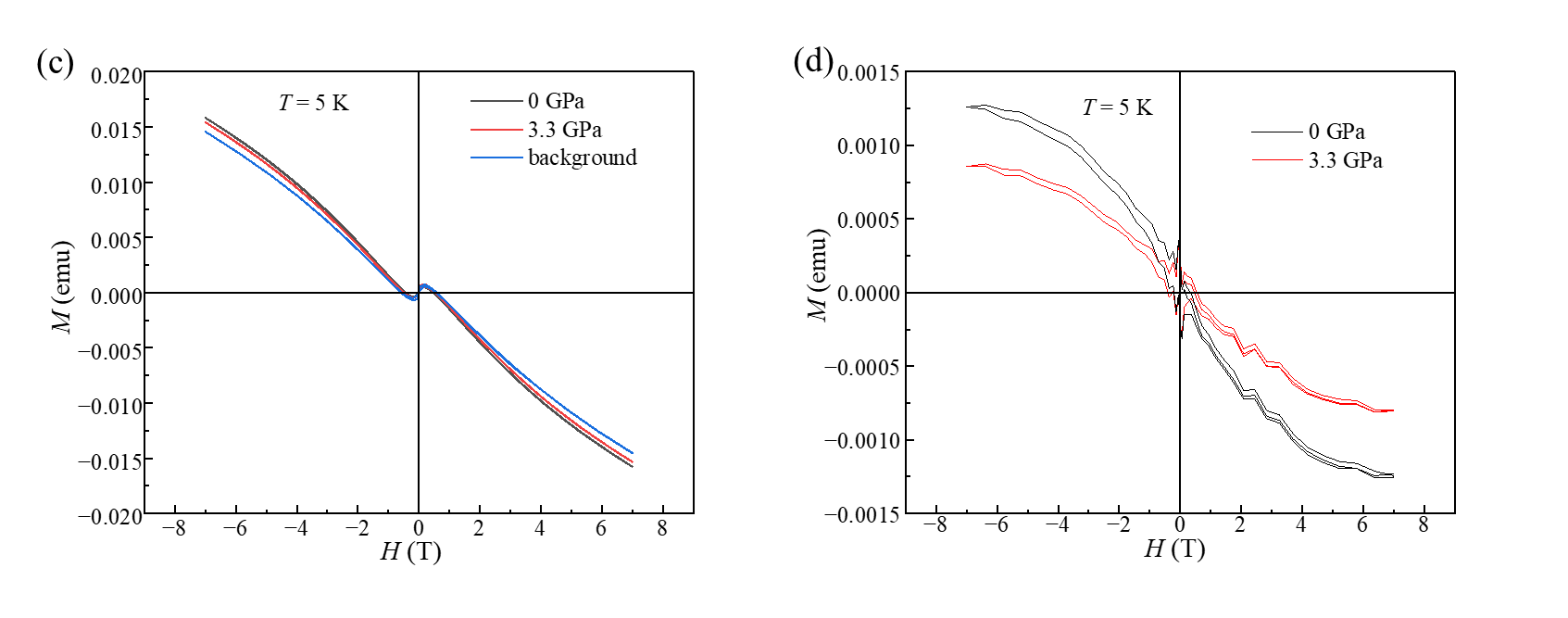
**

**Figure S5.** Raw data of temperature-dependent magnetization, *M*(*H*), measured with the piston-cylinder cell with cooling field of (a) 0.1 T and (b) 7 T. *M*_+_ and *M*_-_ are positive and negative remnant magnetizations. *H*_+_ and *H*_-_ are right and left coercive fields. (c) Raw data of *M*(*H*) measured with DAC at 5 K under pressure of 0 and 3.3 GPa, along with *M*(*H*) of empty DAC as background signal. (d) Corresponding *M*(*H*) of 0 and 3.3 GPa after subtracting the background magnetization.

**Figure S6**


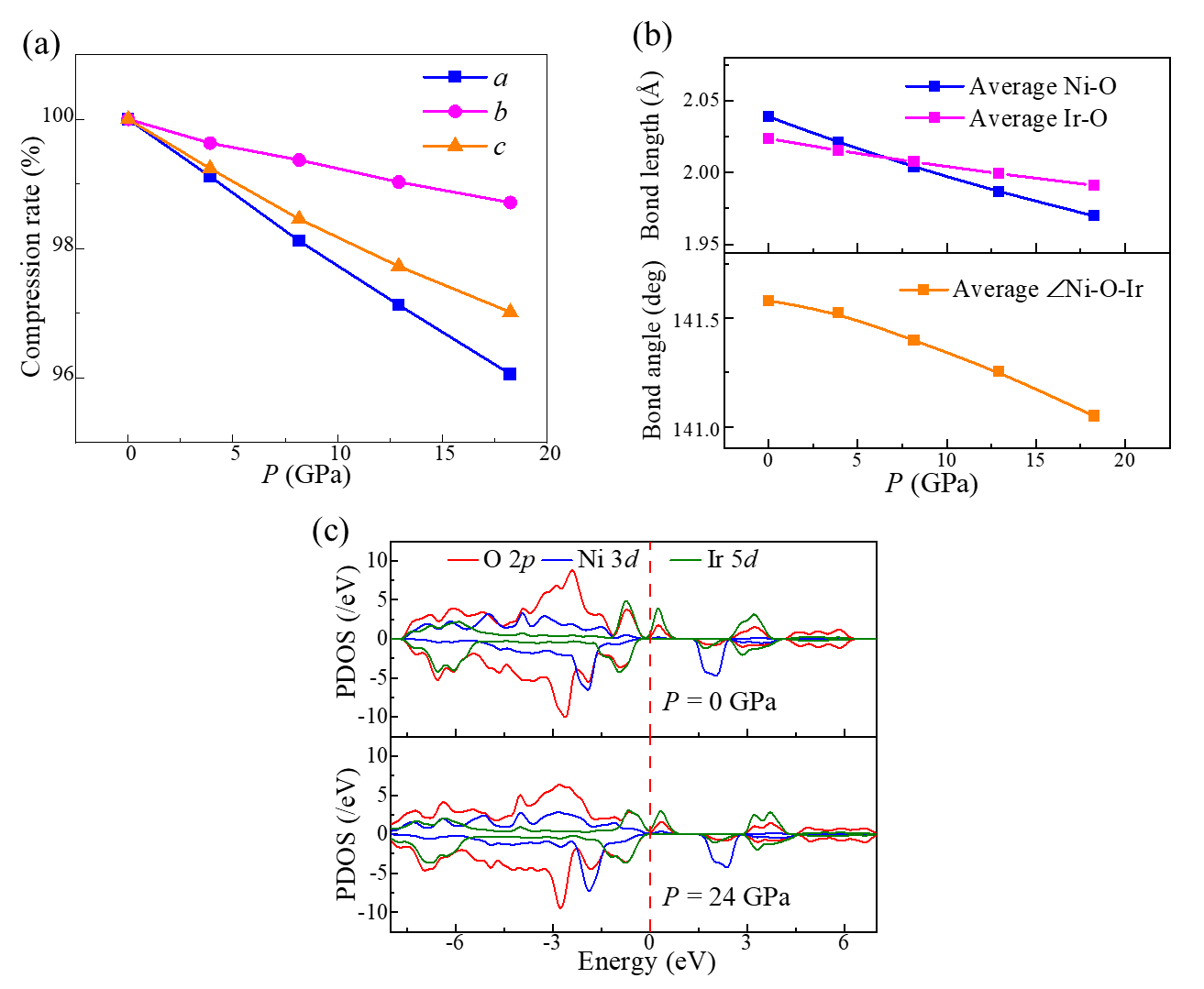


**Figure S6. Calculated lattice parameters and physical properties of YNIO.** (a)The changes in compression rate of the unit cell parameters under different pressures. (b) Calculated average Ni–O and Ir–O bond lengths and average ∠Ni–O–Ir of YNIO as functions of pressure. (c) PDOS of YNIO at *P* = 0 and 24 GPa. Note that *V*e_q_(24 GPa) = 0.9*V*e_q_(0 GPa). The Fermi level is set to 0 eV as indicated by a red dashed line.

**Table S1**

**Table S1.** Structural parameters of Y_2_NiIrO_6_ as determined from Rietveld refinements at varying pressures. Ni-Ir antisite ratio is 7.5%.

| Pressure (GPa) | 0 | 0.56 | 3.0 | 6.0 | 9.3 | 13.0 | 17.0 |
| --- | --- | --- | --- | --- | --- | --- | --- |
| *a* (Å) | 5.2662 | 5.2656 | 5.2454 | 5.2169 | 5.1900 | 5.1622 | 5.1284 |
| *b* (Å) | 5.6850 | 5.6857 | 5.6853 | 5.6785 | 5.6707 | 5.6654 | 5.6592 |
| *c* (Å) | 7.5858 | 7.5844 | 7.5624 | 7.5353 | 7.5053 | 7.4778 | 7.4491 |
| *β* (°) | 90.15 | 90.12 | 90.44 | 90.52 | 90.43 | 90.33 | 90.60 |
| *V* (Å^3^) | 227.11 | 227.07 | 225.52 | 223.22 | 220.88 | 218.69 | 216.18 |
| Y | 0.0234,  0.0788,  0.2466 | 0.0194,  0.0756,  0.2489 | 0.0132,  0.0809,  0.2472 | 0.0124,  0.0845,  0.2482 | 0.0120,  0.0826,  0.2454 | -0.0025,  0.0835,  0.2465 | 0.0043,  0.0856,  0.2488 |
| Ni | 1/2, 0 ,1/2 | 1/2, 0 ,1/2 | 1/2, 0 ,1/2 | 1/2, 0 ,1/2 | 1/2, 0 ,1/2 | 1/2, 0 ,1/2 | 1/2, 0 ,1/2 |
| Ir | 1/2, 0 ,0 | 1/2, 0 ,0 | 1/2, 0 ,0 | 1/2, 0 ,0 | 1/2, 0 ,0 | 1/2, 0 ,0 | 1/2, 0 ,0 |
| O_1_ | 0.1869,  -0.1878,  0.0568 | 0.1877,  -0.1895,  0.0564 | 0.1850,  -0.1870,  0.0572 | 0.1870,  -0.1905,  0.0592 | 0.1832,  -0.1910,  0.05840 | 0.1824,  -0.1879,  0.0620 | 0.1846,  -0.1875,  0.0618 |
| O_2_ | 0.6196,  -0.0486,  0.2503 | 0.6201,  -0.0489,  0.2500 | 0.62056,  -0.0451,  0.2517 | 0.6182,  -0.0493,  0.2513 | 0.6171,  -0.04855,  0.24955 | 0.62035,  -0.04851,  0.24967 | 0.6204,  -0.0483,  0.2497 |
| O_3_ | 0.3197,  0.3052,  0.0611 | 0.3193,  0.3061,  0.0621 | 0.3193,  0.3022,  0.0625 | 0.3222,  0.3031,  0.0639 | 0.3262,  0.3085,  0.0624 | 0.3263,  0.3112,  0.0622 | 0.3268,  0.3118,  0.0668 |
| Average *l*_Ni-O_ (Å) | 2.0419 | 2.0503 | 2.0475 | 2.0423 | 2.0397 | 2.0322 | 2.0292 |
| Average *l*_Ir-O_ (Å) | 2.0289 | 2.0228 | 2.0173 | 2.0111 | 2.0105 | 2.0091 | 1.9994 |
| Average ∠Ni-O-Ir (°) | 141.885 | 141.670 | 141.615 | 141.457 | 141.015 | 140.288 | 139.980 |

**Table S2**

**Table S2.** *H*_C_, *H*_EB_ and *M*_shift_ at different pressures and cooling fields. *H*_C_ = 1/2(*H*_+_+*H*_-_), *H*_EB_ = 1/2(*H*_+_-*H*_-_), *M*_shift_ = 1/2(*M*_+_-*H*_-_)

| Cooling field (T) | Pressure (GPa) | *H*_C_ (T) | *H*_EB_ (T) | *M*_shift_ (emu) |
| --- | --- | --- | --- | --- |
| 0.1 | 0 | 4.35 | 0.643 | 0.0050 |
|  | 0.8 | 4.40 | 0.790 | 0.0057 |
| 7 | 0 | 4.45 | 0.823 | 0.0095 |
|  | 0.8 | 4.56 | 1.064 | 0.0106 |

**Table S3**

**Table S3.** Energy differences between ferromagnetic (FM) and antiferromagnetic (AFM) states with varying *U*_eff_ values for Ni and Ir. ∆*E* = *E*_FM_ – *E*_AFM_

| *U*_eff_ (eV) | ∆*E* (eV) |
| --- | --- |
| *U*_eff_(Ni) = 4.0, *U*_eff_(Ir) = 2.6 | 1.6×10^-5^ |
| *U*_eff_(Ni) = 3.0, *U*_eff_(Ir) = 2.6 | 3.3×10^-5^ |
| *U*_eff_(Ni) = 3.5, *U*_eff_(Ir) = 2.6 | 4.2×10^-5^ |
| *U*_eff_(Ni) = 5.0, *U*_eff_(Ir) = 2.6 | 1.3×10^-5^ |
| *U*_eff_(Ni) = 4.0, *U*_eff_(Ir) = 0.6 | 8.6×10^-5^ |
| *U*_eff_(Ni) = 4.0, *U*_eff_(Ir) = 1.6 | 3.2×10^-5^ |
| *U*_eff_(Ni) = 4.0, *U*_eff_(Ir) = 2.1 | 9.9×10^-5^ |
